# Quasispecies-like behavior observed in catalytic RNA populations evolving in a test tube

**DOI:** 10.1186/1471-2148-10-80

**Published:** 2010-03-23

**Authors:** Carolina Díaz Arenas, Niles Lehman

**Affiliations:** 1 Department of Chemistry, Portland State University, PO Box 751, Portland, Oregon, 97207, USA

## Abstract

**Background:**

During the RNA World, molecular populations were probably very small and highly susceptible to the force of strong random drift. In conjunction with Muller's Ratchet, this would have imposed difficulties for the preservation of the genetic information and the survival of the populations. Mechanisms that allowed these nascent populations to overcome this problem must have been advantageous.

**Results:**

Using continuous *in vitro *evolution experimentation with an increased mutation rate imposed by MnCl_2_, it was found that clonal 100-molecule populations of ribozymes clearly exhibit certain characteristics of a quasispecies. This is the first time this has been seen with a catalytic RNA. Extensive genotypic sampling from two replicate lineages was gathered and phylogenetic networks were constructed to elucidate the structure of the evolving RNA populations. A common distribution was found in which a mutant sequence was present at high frequency, surrounded by a cloud of mutant with lower frequencies. This is a typical distribution of quasispecies. Most of the mutants in these clouds were connected by short Hamming distance values, indicating their close relatedness.

**Conclusions:**

The quasispecies nature of mutant RNA clouds facilitates the recovery of genotypes under pressure of being removed from the population by random drift. The empirical populations therefore evolved a genotypic resiliency despite a high mutation rate by adopting the characteristics of quasispecies, implying that primordial RNA pools could have used this strategy to avoid extinction.

## Background

During the origins and early evolution of life on the Earth, the contemporary notion of well-delineated species was not yet realized. Instead, the adaptive fate of populations of naked molecules would have been more accurately described by the quasispecies model of mutant distributions, which was introduced in the 1970's by Eigen [1-3]. Under an RNA World scenario, the molecules in question were autocatalytically replicating polymers of nucleotides, such as RNA or a chemical equivalent [4,5].

A quasispecies is basically a steady-state dynamic of mutant molecules distributed around a parental genotype, the so-called master sequence, which occupies a central position in the genotypic network space. This dynamic occurs after a sufficient amount of time at high mutational rates, such that the progeny of an individual genotype (the mutant cloud) can be rapidly produced. Importantly, the target of selection is the genotypic distribution as a whole, not single genotypes. Eigen realized that simple genetic entities would gain an evolutionary advantage by a primitive form of group selection in which replication rate deficiencies could be offset by the heightened production of new mutant types [1]. Different genotypes can form mutant clouds of various sizes that can compete for survivorship during the evolution of the population. This can generate a fluctuating equilibrium dynamic as clouds of mutants are replaced by other ones at the interplay of selection and random drift [6].

Although the quasispecies concept was originally intended as a description of molecular replicators cooperating and competing for survival prior to their historical encapsulation in membranes that inexorably linked genotype with phenotype [5,7], it soon was argued that many viral populations evolved with the same dynamic. The first empirical demonstration of quasispecies behavior was produced in 1978 with the Qβ phage [8]. Since then many viral populations have been described as evolving as quasispecies collectives under mutation-selection balance [9-15], although this interpretation has been debated in the case of viruses [7,16]. Moreover, many experiments, particularly by Biebricher and co-workers, have clearly highlighted the power of the quasispecies concept to describe the evolutionary progress of naked RNA molecules in the test tube as they are replicated by error-prone protein polymerases [17-19]. Such experiments confirm some of the key predictions of quasispecies dynamics as outlined by Eigen [5,7]: a cloud of neutral mutations exists that surround a central genotype, selection operates on this cloud as a whole, in small and error-prone populations many specific mutants occur reproducibly (recur), and the greatest amount of genotypic diversity exists just below an error threshold above which information decays into chaos.

To date however, the use of a catalytic RNA in a system capable of forming quasispecies has not been reported. Currently available data are restricted to genomic or genome-fragment RNAs that can carry genetic information but not perform a catalytic function. The aim of the current work is to extend the quasispecies concept back at least as far as to a system in which the catalytic function of an RNA molecule is integral to its own replication process, even if the actual polymerization of nucleotides is carried out by an exogenous polymerase. Here, the continuous evolution (CE) *in vitro *evolution [20] of class I ligase ribozymes is used to create an evolving RNA population that can be tracked over hundreds of generations in a very short time frame. This system has previously been shown to exhibit mutational meltdown [21] of small populations when the mutation rate is limited by the intrinsic error rate of viral polymerases [22]. Now, with the error rate greatly enhanced with the addition of a chemical mutagen (manganese (II) ion) such that prebiotic conditions are better simulated, it is shown that ribozyme populations do indeed display quasispecies-like dynamics while evolving in a test tube.

## Results

### CE experiments

**Figure 1:**
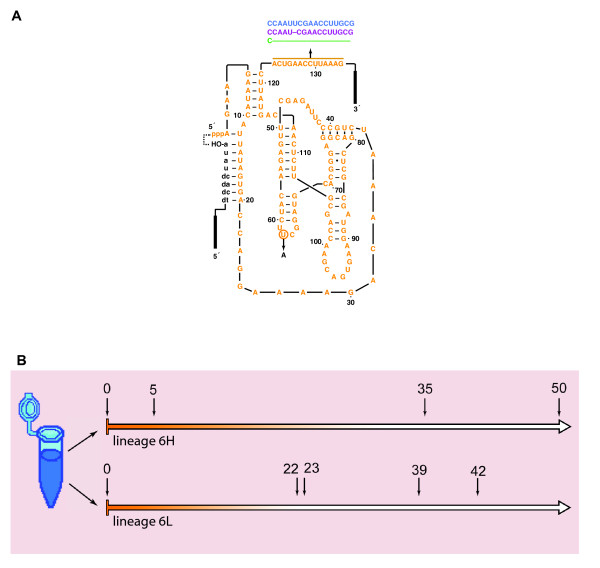
**Ligase secondary structure and bursts sampled from two lineages of continuous *in vitro *evolution**. Two lineages were evolved from a homogeneous population of 100 molecules of B16-19 ligase ribozyme (A). The secondary structure of the B16-19 ribozyme is shown in orange, in which the primer binding site for reverse transcription (3' end) and the 5' end of the promoter for forward transcription (5' end) during CE are denoted by solid rectangles. The ribozyme effects the ligation of an external substrate oligomer (black lowercase letters) that contains the T7 RNA transcriptase promoter sequence, needed for CE, to its own 5' end. The ligase ribozyme catalyzes the attack of the 3'-hydroxyl group of the substrate onto the 5'-α-phosphate of the ribozyme (dashed arrow). Mutations that occurred during CE lineage that produced new quasispecies centered around the master sequences are shown, following the color scheme used throughout this paper: MS1 (purple), MS2 (blue), and MS3 (green). All evolved master sequences contain the U62A mutation. Genotypic samples of ligases were taken at various serial transfers in both lineages (B). The lineages were started from a homogeneous population indicated by orange. As time passes, mutated forms arise and accumulate in the population generating an increased diversity, indicated by white. Based on RFLP data [Additional file 1: Supplemental Figure S1] the bursts selected for cloning and sequencing are indicated by small arrows: 5, 35 and 50 in lineage 6H; and 22, 23, 39 and 42 in lineage 6L.

Four clonal 100-molecule populations of the RNA class I ligase ribozyme B16-19 (Figure 1A) were each evolved independently using the CE system. The CE protocol is a means to induce the rapid evolution of ligase ribozymes using Moloney Murine Leukaemia Virus Reverse Transcriptase (MMLV-RT) and T7 RNA polymerase to sustain RNA populations through sequential serial transfers [20,23,24]. Each serial transfer involves roughly three cycles of amplification that produces a rapid proliferation of RNA molecules, and hence is termed "burst" in this paper. The experimental conditions used were the same as in earlier experiments [22,25,26], with the exception that MnCl_2 _was added to the reaction vessel at a final concentration of 40 μM to increase the mutational rate. The *in vitro *per nucleotide error rate of MMLV-RT has been estimated at about 1/30,000 [27], but Mn^2+ ^ions lower the substrate specificity of RNA- and DNA-dependent DNA polymerases both *in vivo *and *in vitro*, resulting in a 6-30-fold higher error rate [28-30]. All four lineages (termed 6E, 6H, 6K, and 6L) were carried out for 50 bursts without a sign of population decay via Muller's Ratchet, and thus a mutational meltdown was never observed [Additional file 1: Supplemental Figure S1]. These results were unlike those of previous data obtained in the absence of MnCl_2_, in which a meltdown was observed at an average of 24.3 bursts [22]. In fact, the data collected here were directly compared to a more extended analysis of one lineage from the previous study (termed 3D) that actually did survive to burst 50 [22].

### Genotypic characterization

To investigate the cause of the extended time to extinction, a preliminary inspection of the populational genetic variability using RFLP was performed. In general, the cDNA in a population at any burst can be amplified via PCR and then genotyped by either RFLP or nucleotide sequence analysis. The fixation, or nearly so, of mutant forms was evidenced in all the lineages at selected bursts. Based on RFLP assays [Additional file 1: Supplemental Figure S2] two lineages (6H and 6L) were selected for a more extensive characterization of the genotypes present in the populations. Genotypes from three or four bursts, respectively, in lineages 6H and 6L (Figure 1B) were cloned and sequenced exhaustively enough to gather a representative sample of the population diversity. In addition, two bursts from the smallest surviving lineage from the previous study [22] without added MgCl_2 _(the 600-molecule lineage 3D) were genotyped for comparison.

### Network analysis

**Figure 2:**
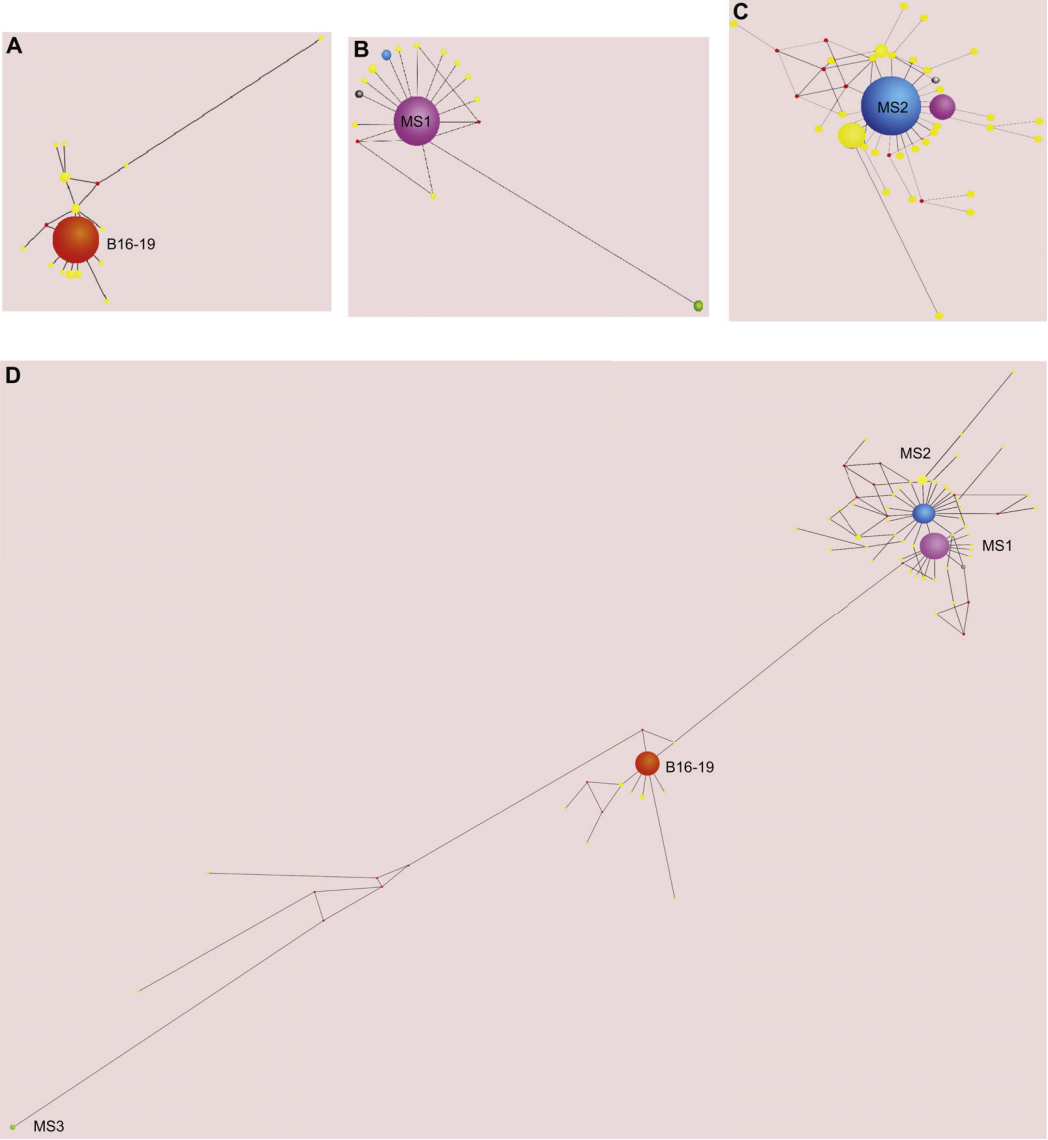
**The quasispecies formed during the evolution of lineage 6H**. Networks among genotypes were constructed using Network v. 4.5.1.0. Different clouds can be observed in each burst evaluated: (A) burst 5; (B) burst 35; and (C) burst 50. Additionally, a consolidated alignment of all sequences in the lineage shows a different network outcome (d). Spheres represent mutants present at different frequencies, denoted by the length of the radius. The color of the sphere is different than yellow if the mutant persists through bursts or recur in a different lineage. The colors are maintained in all the networks drawn. The distance between spheres is representative of the Hamming distance between the sequences. The red spheres are calculated median vectors that represent non-sampled or predicted ancestral individuals [64]. The name of the master sequences in each burst is given as follows: (A) wildtype = B16-19 ligase ribozyme; (B) MS1; and (C) MS2, according to their order of appearance.

**Figure 3:**
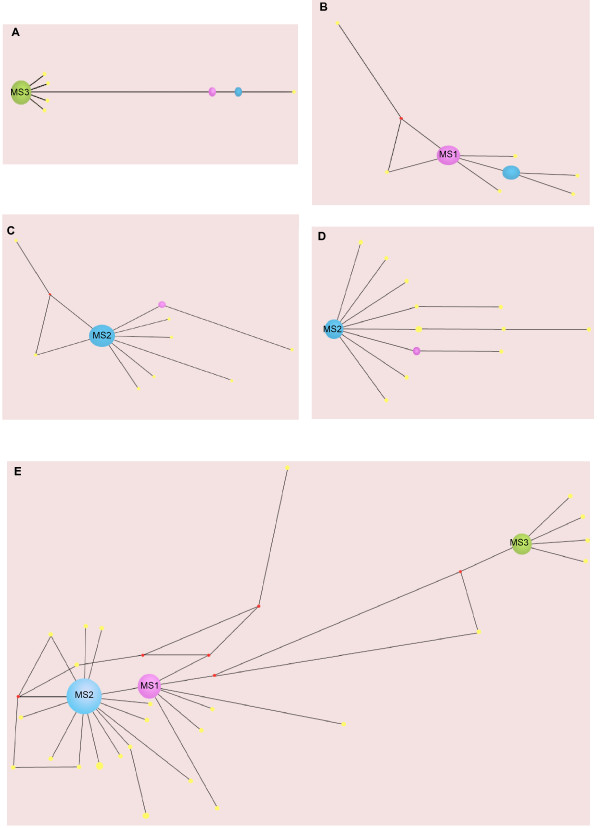
**The quasispecies formed during the evolution of lineage 6L**. As in lineage 6H, different clouds are formed in each burst evaluated: (A) burst 22; (B) burst 23; (C) burst 39; and (D) burst 42. The master sequence names are given according to their order of appearance, as a continuation of the numbering in lineage A. Note that the genotype of the MS3 appeared in the cloud A35 at low frequency, and that MS1 and MS2 are also recurrent from lineage 6H. Spheres represent mutants present at different frequencies denoted by their radii. The genetic relationships between the mutants and the MS are given by their Hamming distances. Only mutants that persist through various bursts or lineages are colored different than yellow, and the color is preserved throughout all the networks. Red spheres are calculated median vectors that represent either ancestral sequences or extant sequences not sampled. A different network outcome is formed from a consolidated alignment of all the sequences in the lineage (E).

Alignment of the nucleotide sequencing data for lineages 6H and 6L showed a trend in the population dynamics in which a majority of the clones have the same genotype, while a minority have slightly different ones. This observation was the first clue that quasispecies behavior may be present in these ligase populations. Phylogenetic networks were drawn (Figures 2 and 3) to find the genetic relationships among the mutants in both lineages, and hence the structures of each putative quasispecies [15]. The structures of these networks show a dynamic characterized by a dominant and centered sequence that is present at a high frequency, around which are located other, less frequent mutant sequences. This population structure is indeed characteristic of a quasispecies, in which the dominant sequence is called the "master sequence" and the surrounding mutants form the mutant cloud [2,3]. These quasispecies are characterized by a relatively close connection among the mutants as indicated by the Hamming distances calculated within bursts (mean = 1.40; mode = 1; min = 1; max = 18) based on the network diagrams (Figures 2 and 3; Table 1). The close connectivity between these mutants is suggestive of a mutational resiliency in the population that is a plausible cause of their observed extended persistence times; they did not go extinct prior to burst 50 unlike all the 100-molecule lineages examined previously [22].

**Table 1:** Summarized characteristics of the quasispecies in CE lineages.

lineage name	3D	3D	6H	6H	6H	6L	6L	6L	6L
burst evaluated	10	50	5	35	50	22	23	39	42
*N*_e _(molecules)	600	600	100	100	100	100	100	100	100
mutagen (MnCl_2_) added?	No	No	Yes	Yes	Yes	Yes	Yes	Yes	Yes
master sequence identity	n/a	n/a	wt	MS1	MS2	MS3	MS1	MS2	MS2
frequency of most common sequence (%)	42	40	77	80	45	68	56	77	62
sample size (sequences)	33	48	102	98	108	41	48	53	42
# of unique genotypes	12	17	17	15	37	9	10	10	13
Shannon Diversity Index	1.96	2.22	1.13	1.01	2.35	1.17	1.50	0.99	1.56
mean Hamming distance	7.2	4.5	2.0	0.95	1.4	1.5	1.2	1.3	1.3

Key: Lineage 3D is from a previous study [22], while lineages 6H and 6L are from this study. The master sequence (MS) names are given by order of appearance, simply 1, 2, *etc*., except in the case of the wildtype (wt), B16-19. The frequency of the MS, the total number of sequences sampled, and the number of unique genotypes was calculated by the Network software, and the Shannon Diversity Index values were calculated using the formula given in the text.

**Figure 4:**
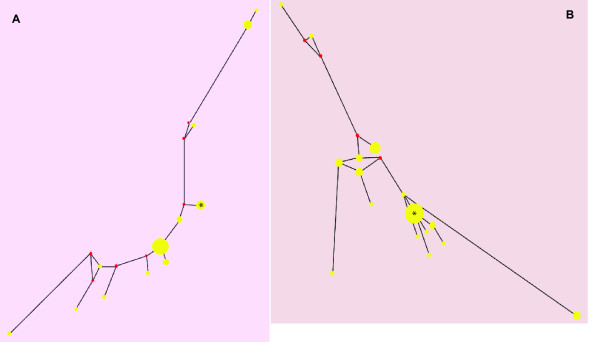
**The genotypic network formed during the evolution of lineage 3D**. Data for this network was obtained by analysis of the smallest surviving lineage evolved without added MgCl_2 _[22]. As in lineages 6H and 6L (Figures 2 and 3), different clouds are formed in each burst, but dominant master species do not appear: (A) burst 10; (B) burst 50. The genotype indicated by the asterisk (*) is the same in both bursts.

For comparison, 33 and 48 clones, respectively, from bursts 10 and 50 of the smallest lineage (3D, ref. [22]) that survived in the absence of added MnCl_2 _were genotyped. Unlike lineages 6H and 6L, the network diagrams of these bursts failed to exhibit a dominant master sequence (Figure 4). Instead, there appeared to be a greater number of less-common genotypes competing for existence, as would be expected under more typical conditions of directional selection and/or random genetic drift. In addition to the network diagrams, one manner in which lineage 3D could be compared to the quasispecies in lineages 6H and 6L is to enumerate the frequency of the most common genotype. In lineage 3D this value did not exceed 42%, while in lineages 6H and 6L this value dropped below 50% only once in seven cases: in burst 50 of lineage 6H (Table 1), where the network diagrams indicated that a transition between one master sequence and another was in progress (Figure 2C). Also the average Hamming distance within the 3D and 3L lineages was 5.60, significantly higher than in 6H or 6L (Table 1; *t*-test; *P *< 0.01).

### Evolutionary dynamics

The observed clouds also showed that there existed a fluctuating dynamic in these populations, as the shape of the clouds -- and gross amount of mutant sequences -- changed from one burst to another (Figures 2D and 3E). These results indicate that each lineage can develop a different dynamic over the course of 50 bursts. It is likely that the high mutation rate used, coupled with the relative stability of a putative quasispecies structure, allows the populations to explore multiple viable alternatives of sequence space during the course of their evolutionary history. In lineage 6H, an early time point (burst 5 out of 50) shows that a quasispecies-like cloud is already formed (Figure 2A), in which the master sequence is still the "wildtype" B16-19, with a frequency of 76%. This cloud was constructed from a sample of 102 clones, which contained 16 different genotypes (Table 1).

The numbers of clones in each burst that needed to be genotyped by nucleotide sequence analysis in order to sample the bulk of unique genotypes present in the population was evaluated by constructing rarefaction plots. Because between 41-108 clones per burst were genotyped (Table 1), and yet the harmonic mean population size of these bursts was ~100 individuals and the frequency distributions of genotypes were highly skewed (see above), these rarefaction analyses indicated that this sampling was exhaustive enough to capture all but the rarest of genotypes. The required number of clones to be inspected was similar when comparing the bursts within the lineages, but different when comparing the two lineages [Additional file 1: Supplemental Table S1]. This result is not surprising, because a quasispecies would be a fairly stable equilibrium dynamic, and the environmental conditions are nearly constant during the CE experiments. It should be noted that, although the population size was ostensibly kept constant at 100 molecules throughout each lineage, the cloning procedure did involve PCR amplification. Therefore, the sampling of genotypes from the population is effectively sampling with replacement of the total diversity. For example, 102 clones from burst 5 of lineage 6H were obtained, but this did not mean that all individuals were sampled. Nevertheless, the observed sample diversity in this burst was estimated at 1.49, as measured by Shannon Diversity Index (*H *= -Σ*p*_*i*_(ln*p*_*i*_) where *p*_*i *_is the frequency of the *i*th clone). Deeper examination of the bursts in this lineage (Figures 2B and 2C) revealed a change in the master sequence identity, frequency, number of genotypes, and Shannon diversity values. In general, the quasispecies formed in each burst presented different identities from each other, but their characteristics are fairly similar (Table 1). This similarity is perhaps the result of the fact that the sequence space available for exploration by the populations is bounded by a unique starting point; they are all genotypically identical at the beginning of the evolution experiment. Therefore, the area of sequence space that can be explored in 50 serial transfers would be relatively short, and the lineages may be not very different in their diversity values.

Genotypes that are present at a higher frequency in one burst can become a master sequence at a later burst. Conversely, a master sequence that, having once been displaced, was never observed to come back to high frequency in the population. For example, in lineage 6H, the following transition can be observed in the master sequence identity and frequency: burst 5, B16-19 (86%); burst 35, MS1 (91%); burst 50, MS2 (48%). This dynamic of master sequences being displaced by one another resembles that of clonal interference, in which advantageous mutants have to compete for resources and some get displaced [31-33]. A network drawn by combining the sequences of all bursts inspected in lineage 6H (Figure 2D) shows the fluctuation of the equilibrium dynamic of the quasispecies in its evolutionary history.

In lineage 6L, four bursts were evaluated (Figure 3). These specific bursts were chosen because the RFLP analysis detected genotypic transitions near these time points [Additional file 1: Supplemental Figure S2]. The dynamic of the lineage is similar to that of lineage 6H, in that different quasispecies clouds emerge and evolve through time. Two successive bursts (#22 and #23; Figure 1B) reveal that this fluctuation can occur in a relatively short time (Figures 3A and 3B). Interestingly, some of the master sequences that appear during this lineage (Figures 3B, C, and 3D) are the same as were observed in lineage 6H (Figures 2B and 2C). These results suggest that some quasispecies may develop a stronger mutant coupling than others that enables them to recurrently out-compete other quasispecies present during the lineages evolution. Similar to the pattern in lineage 6H, the characteristics of the quasispecies change over the course of the evolutionary history as indicated by the master sequence identity, frequency and number of genotypes, and diversity values calculated (Table 1). These dynamics of staggered dominant genotypes that fluctuate as the population evolves (Figure 3E) may be a reflection of the interplay of Darwinian selection and random genetic drift acting on the quasispecies.

Other than the genotype used to seed these experiments (B16-19 = the "wildtype"), the genotypes that appeared in lineages 6H and 6L were not identical to the dominant genotypes that appeared in lineages evolved without added mutational pressure [22]. Some specific mutations, such as the U62A "insurance" mutation [22], did appear recurrently in the quasispecies in lineages 6H and 6L, but the composite genotypes seen in this study were not those observed previously. In particular, the "immunity" mutation (a change from CUGAACCUUA to AAUCG at positions 123-132), which conferred resistance to mutational meltdown in small population sizes in the absence of MnCl_2_, was not seen in the current study, although short insertions, deletions, and rearrangements were common in the last 10-12 nt at the 3' end of the ligase (Figure 1), and specific mutations in this region appeared in more than one quasispecies. Recurrence of mutations is actually a predicted characteristic of quasispecies [5], thus providing additional support for this interpretation of these data.

**Figure 5:**
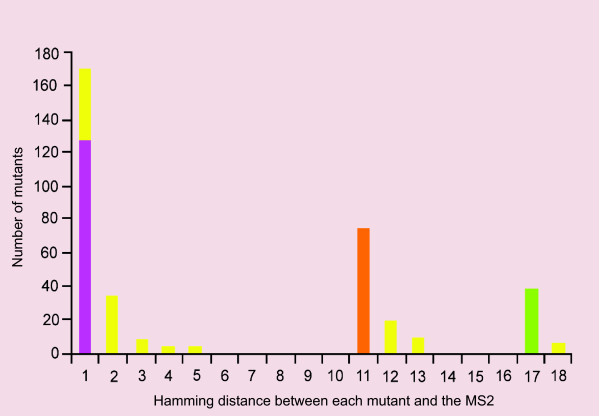
**Frequency histogram of Hamming distance values between MS2 and all other mutants**. Color codes are the same as previous figures. The *x*-axis corresponds to the Hamming distances calculated between each mutant type and the sequence of the MS2, and the *y*-axis correspond to the frequency at which mutants with that specific Hamming distance are present in the populations (sum of lineages 6H and 6L). Note that overall, the most frequent mutants have a Hamming distance of one compared to MS2, and most of them constitute the MS1 (127 out of 170). The groups with large Hamming distance values are B16-19, used to start the experiment (and once replaced did not come back), and MS3 (the least recurrent of all the MS). The majority of the mutant sequences that have appeared at one burst have recurred at another burst (61%), mainly as a master sequence.

**Figure 6:**
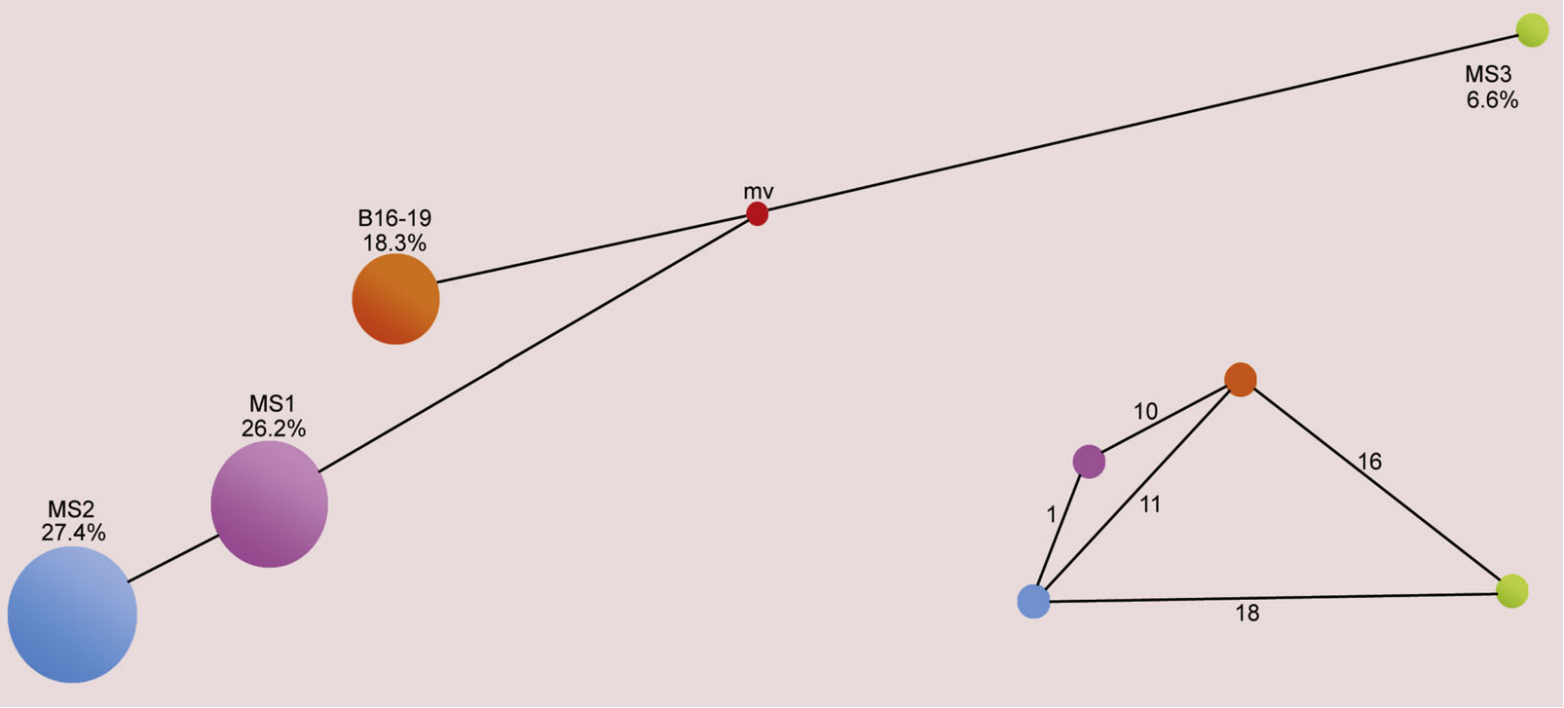
**A simplified network showing the relationship between all the master sequences observed**. Sphere size and color represent the identity and frequency of the master sequences with data gathered from all the burst in both lineages 6H and 6L. Each cloud is labeled with the name and the frequency taking into account all the data. The inset shows the Hamming distances between all the master sequences.

In general, from all the networks drawn, it was observed that the number of mutations that appeared in more than one burst and/or lineage constituted 61% of the total number of sequences explored, and the great majority of these mutants became part of master sequences during the lineages' evolutionary history (green, purple and blue spheres in the quasispecies networks of Figures 2 and 3). The fact that most of the mutants that have evolved in these lineages were able to persist in time could be a consequence of the mutational resiliency that evolved owing to the short Hamming distance values (Figure 5). Most of these recurrent mutations belonged within the master sequences (Figure 6). In lineage 6H, the initial change in master sequences from B16-19 to MS1 implies ten changes, and the further change from MS1 to MS2 implies one change (Figure 6, inset). Similarly, in lineage 6L, the initial change from B16-19 to MS3 implies sixteen changes, but further changes in the master sequence transitions are seventeen and one (Figure 6, inset). The Hamming distance values are generally low (in the range of tens), eighteen being the maximum value. The distance between the two most recurrent master sequences is actually only one, and these master sequences (MS1 and MS2) are the most representative in time and sequence space (54% frequency out of the total). The Hamming distance values, in addition to the high frequency of recurrent mutations, support the idea of a form of mutational robustness [34-36] evolving in the system through a quasispecies behavior in ligase populations evolved *in vitro*, although a formal test of this will require a comparison of fitness values.

## Discussion

"What is a quasispecies?" is the exact title of at least two papers [37,38]. At times this phenomenon has clearly been difficult to detect and apply to real populations with absolute certainty. However the defining characteristics of a quasispecies, as described by Eigen, unequivocally include a spectrum of closely related genotypes and a population that is struggling to survive under a relatively high error rate. These features seem to apply to primordial collections of RNA near the origins of life, and thus prompted the current study. Here, clonal 100-molecule populations of B16-19 ligase ribozymes were evolved using the continuous *in vitro *evolution (CE) method [20] and the relatively error-prone MMLV-RT. In particular, MnCl_2 _was added to the reaction vessel to increase the error rate of protein enzymes. Populations evolved under these conditions did not show a shortened extinction time, as was observed previously when mutational meltdown conditions prevailed under only a weak mutational pressure of no added MnCl_2 _[22]. The data suggest that this is a consequence of the advent of quasispecies in these ribozyme populations.

Quasispecies behavior has never before been demonstrated during *in vitro *evolution experiments with catalytic RNA. Other *in vitro *experiments with ribozymes have shown either convergence on a phenotype or recurrence of a genotype or motif, but not the type of dynamic of quasispecies that we are documenting here. For example, Yingfu Li and colleagues studied how the composition of a population of RNA-cleaving DNAzymes changed over time in response to selective pressures acting on the phenotype [39,40]. Similar to the findings reported here, they found a dynamic fluctuation in the structure of the population. Many sequence classes peaked in frequency at different rounds of selection, but in this case, one class appeared to consistently maintain a high frequency. It will be interesting to explore the population structure that these DNAzymes would adopt if the mutation rate of the replication were increased. Perhaps mutational coupling may arise in these molecular populations as well. Another classic study of interest was the evolution of the RNA variant V2 of the Qβ virus performed by Orgel and co-workers [41]. In this case, the mutagen ethidium bromide (EthBr) was added to the reaction vessel during the serial transfers. RNAs resistant to EthBr evolved and adapted to increasing drug concentrations. However in this case, in contrast to the CE/Mn^2+ ^mutagenesis experiments presented here, the mutagen has a direct effect on the RNA structure, and therefore selection favored variants that mutated away the EthBr-binding sites, and a single "winner" emerged. Examples of experiments in which there is a convergent to a relatively more efficient solution for the population have been reviewed [23,42-45]. Other *in vitro *evolution studies have been initiated from pools of limited diversity but in which a recurrent solution would appear upon independent trials. This was the evolution dynamic that gave rise to the class I ligase [26], and the group I ribozymes [46], but quasispecies-like behavior was not adopted by any of those populations.

The current study employed the ribozyme B16-19, which is highly proficient in catalyzing the ligation reaction necessary for the CE to occur [47,48], and posses an efficient folding into an active conformation [23,25,26]. These characteristics locate this genotype on the top of a high fitness peak [23,26]. Thus, the mutation accumulation that occurred during each serial transfer can cause structural changes in the individuals, which in turn can further cause a decrease in the mean fitness of the population via Muller's Ratchet and random drift. The population can then fall into a fitness valley and become extinct. The addition of Mn^2+ ^to the reaction vessels increases the mutation rate of the replication process and consequently it can alter the equilibrium distribution of the population [6]. Quasispecies behavior sustains this shift in equilibrium distribution by a mutational coupling, allowing the population to stay extant in spite of the strong random drift and the ostensible lack of recombination in the population, as was observed recently in empirical populations of viroids [13].

The shift in the equilibrium distribution is possible because of two main reasons, first stated and then explained.

(1) Catalytic RNA sequences such as ligase ribozymes posses the property of buffering mutations through epistatic interactions between secondary structure arrangements [35,49]. These arrangements strongly stabilize the structure and thus a broader range of mutations will have a neutrally selective effect, hence relaxing the error threshold [49,50].

(2) The fitness of each genotype in the population is normalized with the total number of genotypes in the system (assuming single locus theory applies). Thus, the proportional contribution of each genotype to the total fitness decreases as the number of genotypes increases [51].

The high degeneracy observed in ribozyme genotype-to-phenotype maps insures that the majority of point mutations are neutral [34,35]. In spite of this, the mutational buffering of secondary structure epistatic interactions can only favor the fitness of the lower class mutants (*e.g*., low Hamming distance values). The genetic load generated in higher-class mutants will likely disrupt secondary interactions and the stability of the individual ligases. Oddly enough, an increase in the mutational rate does not cause a proportional increase in the genetic load, and therefore the population does not become extinct at a faster pace. What could be happening in this case is that mutants of lower class emerge quickly, generating a wide low-mutant class in the early evolutionary pathway of the population. These mutants have a short Hamming distances and thus probably similar fitness values. Wilke [51] observed that the first couple of replication cycles mostly determine fixation or extinction for an invading sequence, or perhaps for a group of close connected sequences, such as the quasispecies mutant cloud. The major contribution to the fixation probability comes from the connectivity matrix of the local genetic neighborhood of the invading sequence (or mutant class); sequences farther away on the neutral network that are poorly connected become relatively unimportant, and may be drawn out of the population by genetic drift and mutation selection balance.

The level of connection in the matrix is determined by the Hamming distance values of the mutants in the network. Genotypes that are closely connected by short Hamming distances are closely related in the sense that they can rapidly (*e.g*., in a few generations) be regenerated from one another in the eventual case of being removed from the population by random drift. In contrast, poorly connected genotypes (*e.g*., only distant relatives) will have a slow recovery into the population, if at all. In this scenario, because mutants with short Hamming distance may have close fitness values, individual sequences are not essential for the survival of the population, rather the group of close-connected individuals with mutational robustness [13,52,53]. Therefore, the quasispecies cloud itself is being the target of selection [6] and not the individual sequences. This process is analogous to the manner in which kin selection operates in animal societies [54,55]. Ribozyme populations therefore can -- by means of indirect reproduction effects -- evolve a mutational robustness [57], a behavior that empowers selection with an advantage relative to other evolutionary forces (*e.g*., the strength that random drift has in populations of small effective sizes). This genotypic malleability allows the population to avoid a mutational meltdown, and stay extant.

These results -- of ligase RNA molecules capable of forming population structures in which cooperation is more beneficial than competition -- suggest that altruistic behavior (*e.g*., cooperation) is an advantageous feature to ensure survival of populations during the RNA world [56], when the population size were small, when the mutational rate was high, and when random genetic drift had strong effect, conditions that certainly prevailed on the prebiotic Earth [52,58]. Additionally, quasispecies have an organization structure with the properties proposed by Kaufmann [59] to be necessary for the origin and preservation of genetic information. In this structure, the closely connected cloud of the quasispecies can serve as an information-preserving core, and the distantly surrounding genotypes as ideal targets for random genetic drift because they are less frequent and the information loss through them does not negatively impact the survival of population. According to Kauffman [60], organized systems may have arisen as a consequence of the property of some elements to establish different levels of connectivity among each other. The highly interconnected elements can create organizational cores able to preserve the information relevant to survival of the system (*e.g*., autocatalytic function). In contrast, less interconnected elements can serve as a reservoir of mutations without a detrimental effect on this information. Thus, during the ancient acellular times at the biogenesis on the Earth, the assemblage of information cores, perhaps in the form of quasispecies clouds, may have provided the necessary route to increase population sizes, and allow enough time for information to mature into more sophisticated functions necessary for a cellular type of life.

## Conclusions

The quasispecies is a population structure typically formed at high mutation rates that allow the mutants to stay closely connected and thus be easily regenerated from one another even if lost from the population through random genetic drift. This behavior empowers selection relative to other evolutionary forces. Consequently, information relevant to the survival of the population can be stored in a close-knit network of mutants and not in the individuals. It is likely that such a population structure would have greatly benefited primordial pools of nascent RNA molecules on the early Earth. Instead of relying on the fortuitous advent of specific self-replicating genotypes, the RNA World would have the luxury of swarms of quasispecies evolving over time, buffered against extinction through informational decay, as theorized by Eigen [1].

## Methods

### RNA Preparation

B16-19 ligase ribozymes were freshly prepared by transcription of PCR DNA of B16-19 clones obtained in a previous *in vitro *evolution experiment [25]. The RNA transcripts were purified by PAGE. The concentrations of the RNAs obtained after gel purification were measured by UV spectroscopy at 260 nm. A dilution series was then performed to obtain the desired concentration of 100 molecules in the 8.20 μL aliquot used to seed the evolution experiments.

### Continuous *in vitro* evolution

Ligase ribozyme populations were evolved using the continuous *in vitro *evolution methodology [20,25,26,61]. To summarize, 2.03 × 10^-8^nM B16-19 ligase (100 molecules) were incubated with 64 pmol of the substrate oligo S-163 (5'-CTTGACGTCAGCCTGGAC*TAATACGACTCAC****UAUA***-3' = a DNA/RNA chimera, with ribonucleotides in boldface letters, and the T7 promoter in italics), 50 pmol of TAS 1.23 primer for reverse transcription (5'-GCTGAGCCTGCGATTGG-3'), 250 units of MMLV reverse transcriptase (United States Biochemicals, Cleveland), 50 units T7 RNA polymerase (Ambion, Austin, TX), 5 nmol each dNTP, 50 nmol each rNTP, 25 mM MgCl_2_, and 40 μM MnCl_2_, in a reaction buffer with 50 mM KCl, 30 mM 4-(2-hydroxyethyl)piperazine-1-propanesulfonic acid (EPPS), pH 8.3. The 25 μL reaction mix was incubated at 37°C for exactly 22 minutes, at the completion of which the reaction was stopped by the addition of 981 μL of water. An 8.2 μL aliquot was taken from the dilution tube and used to seed the next reaction cycle of the continuous evolution process, thereby preserving a constant harmonic mean of the population size [61].

### Population assessments

(1) The survival of the evolved populations was surveyed through PCR amplifications of all the bursts used to seed a reaction cycle. The PCR products were electrophoresed through 2% agarose gels containing ethidium bromide. Visualization of the gels by trans-illumination allowed the identification of a correctly band size when the population remained alive.

(2) Preliminary genetic variability was evaluated by RFLP using the restriction enzymes employed in the previous publication [22]. In the populations where genotypic variability was detected, a more extensive genotypic characterization was done, as described in the following section.

### Genotypic characterization

Bursts were chosen for in-depth sequence analysis based on whether they displayed variability by RFLP and/or on a general desire to examine early, middle, and later bursts of 50-burst lineages. In one case (lineage 6L), two adjacent bursts were analyzed because the RFLP analysis suggested a rapid genotypic shift at that time. Specific bursts of the evolving populations with genotypic diversity were cloned using the CloneJet™ PCR Cloning Kit (Fermentas, Maryland) and *E*. *coli *competent cells (Invitrogen, San Diego). Between 60-120 colonies per burst were chosen completely at random for genotyping. Colony PCR was used to isolate the insert from single clones and further sequencing was done with BDT v3.1 chemistry. The sequences were aligned with ClustalX 2.0.11 software; the alignments were edited with BioEdit sequence alignment editor v7.0.9.0 (Tom Hall, Ibis BioSciences, Carlsbad) and the chromatogram viewer FinchTV v1.4 (Geospiza Inc., Washington).

To estimate if the number of clones sampled in each selected burst contained all the unique genotypes present in the population, rarefaction plots were constructed. Here, the pool of known genotypes is entered into the computer and then drawn in a random order by a computer (using random numbers [62] imported into Microsoft Excel). Then a plot is made of total new genotypes found as a function of total number of genotypes sampled, following the method used in reference [63]. From averages of these plots, non-linear curve fitting was performed using Origin Pro v8.0 software (OriginLab Corp, Massachusetts) to give the expected asymptotes, which are estimates of the theoretical total number of genotypes present in the population [64].

### Phylogenetic network mapping

For each lineage, all the genetic variants that were detected were aligned using DNA alignment v1.3.0.1 (Fluxus Technology Ltd.) and plotted together using the median-joining method [65] implemented in NETWORK v4.5.1.0 software [66].

## Competing interests

The authors declare that they have no competing interests.

## Authors' contributions

NL designed the project; CDA helped in design details, performed the experiments, and processed the data; NL and CDA discussed the data implications and wrote the manuscript.

## Supplementary Material

Additional file 1 Figure S1 - Comparison of two lineages of ligases evolved at different environmental chemistries; Figure S2 - Mutants detected with TaqαI restriction enzyme; Table S1 - Summary data gathered from the rarefaction plots. Click here for file
