# Supplementary material for: Quasispecies-like behavior observed in catalytic RNA populations evolving in a test tube
Source: BMC Evol Biol. 2010 Mar 23;10:80. doi: 10.1186/1471-2148-10-80 (PMC2850355; doi:10.1186/1471-2148-10-80)
Supplement: Additional file 1 — Figure S1 - Comparison of two lineages of ligases evolved at different environmental chemistries; Figure S2 - Mutants detected with TaqαI restriction enzyme; Table S1 - Summary data gathered from the rarefaction plots. [file 1471-2148-10-80-S1.DOC]

**Supplementary Data**

**Quasispecies behavior observed in catalytic RNA populations evolving in a test tube**

Carolina Díaz Arenas and Niles Lehman

MnCl_2_ added


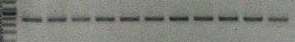


50

48

46

44

42

40

49

47

45

43

41

No MnCl_2_ added


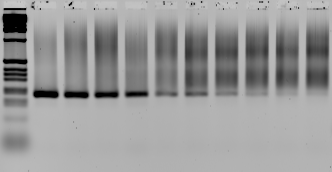


18

15

13

11

9

16

14

12

10

17

### Figure S1 – Comparison of two lineages of ligase ribozymes evolved at high and low mutational pressure

Populations evolved at a high mutation rate, with 0.04 mM MnCl_2_ added to the reaction vessel during continuous evolution *in vitro* (top), in comparison with a population evolved in previous experiments at a low mutation rate with no MnCl_2_ added (bottom) [22]. The presence of the PCR band indicates the survival of the population through time. The lineage evolved in the absence of MnCl_2_ clearly shows the extinction of the population around burst 17, as indicated by lack of a discrete band, as a consequence of mutational meltdown.


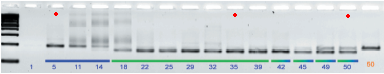

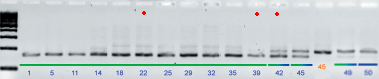


Lineage 6H

Lineage 6L

### Figure S2 – Mutants detected with *Taq*αI restriction enzyme

Numbers in blue are the bursts of the population history that were evaluated; numbers in orange correspond to negative controls (no restriction enzyme added). The upper band in each gel corresponds to the “wildtype” (B16-19) that does not contain a *Taq*αI restriction site, while the lower band corresponds to a mutant form that is digested. Note that in lineage 6H the population size in burst 1 fell just below the PCR detection limit, while in lineage 6L, the *Taq*αI restriction site has been already purged from the population by the end of burst 1. The blue, green and gradient lines help visualize when a polymorphic form become fixed. Red dots indicate the bursts used for nucleotide sequencing and cloning analysis; burst 23 for lineage 6L is not shown in this picture. Two additional lineages (6E and 6K) were also evolved and survived to burst 50, but this restriction enzyme did not detect the same degree of polymorphisms as seen in lineages 6H and 6L. This does not mean that 6E and 6K did not form quasispecies, only that the *Taq*αI screen suggested that 6H and 6L would be better candidates for in-depth nucleotide-sequence analysis. In fact their survival to burst 50 despite their small population size and the added MnCl_2_ would suggest that they were also quasispecies.

### Table S1 – Summary data gathered from the rarefaction plots

The numbers of individuals that needed to be analyzed by direct nucleotide sequencing in order to detect most, if not all, of the genotypic diversity in the population were calculated from the asymptotes of rarefaction plots [61,62]. Note that the amount of individuals sequenced in each burst exceeds in all cases the calculated numbers, hence the samples gathered are representative of the actual number of unique genotypes present in the population.

| Quasispecies | Number of sequenced individuals | Expected number of unique genotypes |
| --- | --- | --- |
| 6H-5 | 102 | 21 |
| 6H-35 | 98 | 27 |
| 6H-50 | 108 | 54 |
| 6L-22 | 41 | 13 |
| 6L-23 | 48 | 13 |
| 6L-39 | 54 | 24 |
| 6L-42 | 45 | 24 |
